# The association of cycle threshold value with clinical features in patients infected with Omicron variant

**DOI:** 10.1016/j.virusres.2025.199565

**Published:** 2025-03-26

**Authors:** Wen Yang, Tao Tao, Jianping Zhang, Yuting Yao, Min Chen, Mingming Liu, Meiying Wu, Wei Lei

**Affiliations:** a Department of Pulmonary and Critical Care Medicine, the First Affiliated Hospital of Soochow University, Suzhou, China; b Department of Pulmonary and Critical Care Medicine, the Affiliated Infectious Diseases Hospital of Soochow University, Suzhou, China; c Department of Pulmonary and Critical Medicine, the Fifth People's Hospital of Suzhou, Suzhou, China; d Department of Pulmonary, the Affiliated Infectious Diseases Hospital of Soochow University, Suzhou, China; e Department of Pulmonary, the Fifth People's Hospital of Suzhou, Suzhou, China

**Keywords:** SARS-CoV-2, Omicron, Clinical features, Ct value, Viral load

## Abstract

This study investigated the correlation between epidemiological and clinical characteristics of patients infected with omicron variants and the cycle threshold (Ct value) for RT-PCR detection. The study population consisted of 115 patients with Omicron infection and the most common symptoms included fever (43.5 %), cough (38.3 %) and sore throat (29.6 %). Laboratory abnormalities were mainly lymphopenia, elevated globulins and elevated blood glucose. Univariate analysis found that older age (*P* < 0.001) and unvaccinated (*P* = 0.003) were associated with low Ct values (high viral load). Multivariate analysis showed that an elevated monocyte count (OR: 3.556; 95 % CI: 1.330–9.503) was associated with low Ct values, whereas being vaccinated (OR: 0.209; 95 % CI: 0.051–0.854) and lower serum sodium (OR: 0.137; 95 % CI: 0.051–0.367) were negatively associated with low Ct values. Studies have shown that factors such as monocyte count, vaccination status and serum sodium correlate with Ct values, suggesting the potential of Ct values as a clinical predictor, which could also provide a valuable reference for clinical decision-making.

## Highlights

• The study examined 115 Omicron-infected patients, finding common symptoms like fever, cough, and sore throat, with lab abnormalities including low lymphocytes and high glucose.
• Older age and lack of vaccination were linked to higher viral loads (lower Ct values).
• Elevated monocyte count, recent vaccination, and reduced sodium levels emerged as potential predictors of Ct values, highlighting their clinical relevance.

## 1 Introduction

By April 2023, the coronavirus disease 2019 (COVID-19) reported as SARS-CoV-2, a severe acute respiratory syndrome coronavirus, had caused an astonishing 761 million infections and 6.8 million fatalities worldwide. SARS-CoV-2 like other RNA viruses is prone to genetic evolution with the development of mutations over time. Based on the recent epidemiological survey, five SARS-CoV-2 VOCs (variant of concern) have been identified: Alpha (B.1.1.7), Beta (B.1.351), Gamma (P.1), Delta, Omicron (Cascella et al., 2022).

Studies report a decreased risk of severe illness and an increasing ability to spread for Omicron, but limited information are available to represent the clinical characteristics and cycle threshold (Ct) values (Chen et al., 2023). Ct values represent the number of amplification cycles required for the target gene to exceed a threshold level. Ct values are therefore inversely related to viral load and can provide an indirect method of quantifying the copy number of viral RNA. However, the effect of Ct values as cliniacal factor is uncertain, which is not only diagnostic tool, but may also contain clinical value.

It has been suggested that lower Ct values may be associated with worse outcomes and that Ct values may be useful in predicting the clinical course and prognosis of patients with COVID-19 (Rao et al., 2020). Although there are differences between the current SARS-CoV-2 compared to the SARS-CoV epidemic, evidence from SARS-CoV indicated that higher viral load was associated with increased need for intensive care and overall worse prognosis (Chu et al., 2004).

Our study focused more on the clinical presentation and impact of Ct values in SARS-COV-2 patients. We aimed to describe the epidemiological, laboratory and radiological characteristics of these patients and tried to explore the relationship between Ct values and clinical features.

## 2 Methods

### 2.1 Study design and data collection

A total of 115 adult patients from the Fifth People's Hospital of Suzhou City between 10 February and 4 March 2022 were included in this retrospective study. Each patient was diagnosed with COVID-19 (Omicron B.1.1.529) and had to comply with the Diagnostic and Therapeutic Regimen for Novel Coronavirus Pneumonia (8th edition) (Diagnosis and Treatment Protocol for Novel Coronavirus Pneumonia (Trial Version 8), 2022). All patients underwent RT-PCR on admission, and blood tests and CT scans were completed within 24 h of admission.Data were collected from electronic medical records and laboratory information systems using standardised forms. The Research Ethics Committee of the Hospital of Infectious Diseases, Soochow University, approved the study.(no.). 202204).

### 2.2 RT–PCR for SARS-CoV-2

Real-time detection of SARS-CoV-2 was achieved by RT-PCR against ORF1ab and nucleocapsid (N) genes. The VOC of SARS-CoV-2 was determined to be Omicron (B).1.1.529) by suzhou CDC. Testing was conducted in a biosafety level II facility.

Virus detection was performed using the Applied Biosystems 7500 Real-Time PCR System, San Diego, USA, according to the instructions of the manufacturer of the BioGerm 2019-nCoV kit (Shanghai, China). The kit is designed to specifically target the open reading frame 1ab (ORF1ab) and nucleocapsid protein (N) genes of SARS-CoV-2. The basic steps of the assay include sample lysis, nucleic acid capture, elution transfer, and multiplex RT-PCR in which analytes are amplified and detected simultaneously. Briefly, 5 μL of extracted RNA was mixed with 12 μL of nucleic acid amplification reaction solution, 4 μL of 2019-nCoV Oligo, and 4 μL of enzyme mixture. A Ct value of <40 for all target genes was considered a positive result.

The Ct value is a measure of the amplification required for the target viral gene to cross a threshold value and is inversely related to the viral load. RT-PCR tests do not measure the viral load, but Ct values offer semi-quantitative assessments of viral RNA concentrations: lower Ct values correspond to higher viral RNA concentrations.When interpreting the results of SARS-CoV-2 RT-PCR, the validity of positive controls using reference materials with known viral copy numbers was confirmed. Also, the kit has negative control and set RNAse P as an internal reference control. The human housekeeping gene target RNAse P (RP) was measured in each sample for use in normalization.

### 2.3 Clinical definitions

The intensity of COVID-19 was measured from mild to severe according to the Diagnostic and Therapeutic Protocol for Novel Coronavirus Pneumonia (8th Edition Trial) (Diagnosis and Treatment Protocol for Novel Coronavirus Pneumonia (Trial Version 8), 2022). Patients with mild clinical symptoms but no signs of pneumonia on imaging were termed "mild", whereas patients with fever and respiratory symptoms accompanied by imaging findings of pneumonia were termed "moderate". "Asymptomatic" was defined as asymptomatic but nucleic acid positive. No diagnosis of severe or critical illness was made.

### 2.4 Statistical analysis

T-test was employed to contrast the mean ± standard deviation of the normally distributed measurement data between groups, while Chi-square and/or Fisher's exact tests were utilized to contrast groups through counts and percentages of categorical variables.Incorporating statistically significant univariate items into binary logistic regression analysis, SPSS software (version 26.0) was employed and P values of <0.05 were taken into consideration.

## 3 Results

### 3.1 Clinical features

The characteristics of the 115 omicron variant confirmed patients were summarized in Table 1. 115 patients consisted of 88 non-moderate cases (22 asymptomatic cases and 66 mild cases) and 27 moderate cases. The median (IQR) age of patients was 37 (29–55) years. 110 (95.7 %) of the patients had a clear history of exposure to positive cases. 38 Comorbidities among patients included hypertension (17[14.8 %]), diabetes (2[1.7 %]), respiratory issues (6[5.2 %]), heart disease (1[0.8 %]), chronic kidney illness (1[0.8 %]), chronic liver illness (2[1.7 %]), malignant tumor (2[1.7 %]), and others (7[6.0 %]). 94 (81.7 %) patients were vaccinated, 8 (9.0 %) had one dose, 50 (53.0 %) had two doses and 36 (38.0 %) had three doses. They all received the vaccine within a year, and 61 (64.9 %) were vaccinated within 6 months. 6 patients had respiratory diseases including pulmonary tuberculosis, bronchiectasis, pulmonary fibrosis, chronic obstructive pulmonary disease, and postoperative lung cancer and 5 of them were moderate cases with image changes in CT (computed tomography) scan.

**Table 1:** Baseline characteristics of patients included in the study.

Baseline characteristics	All patients (*n* = 115)	Non-Moderate type (*n* = 88)	Moderate type (*n* = 27)	*P*
Age (years, median)	37 (29–55)	36 (28–43)	57 (34–68)	<0.001
Gender				
Male	62 (53.9 %)	48 (54. 5 %)	14 (51. 8 %)	0.829
Female	53 (46.0 %)	40 (45. 4 %)	13 (48. 1 %)	
Smoking history	13 (11.3 %)	10 (11. 4 %)	3 (11. 1 %)	0.971
BMI	23.6 (21.6–25.3)	23.5 (21.6–25.5)	24.0 (20.0–24.4)	0.644
Vaccination	94 (81.7 %)	74 (84. 1 %)	20 (74. 1 %)	0.239
Epidemiological history				
Yes	110 (96.0 %)	84 (95. 4 %)	26 (96. 2 %)	0.851
No	5 (4.0 %)	4 (4. 5 %)	1 (3. 7 %)	
Comorbidity				
Hypertension	17 (14.8 %)	11 (12. 5 %)	6 (22. 2 %)	0.213
Diabetes	2 (1.7 %)	2 (2. 2 %)	–	–
Respiratory diseases	6 (5.2 %)	1 (1. 1 %)	5 (18. 5 %)	<0.001
Heart-related diseases	1 (0.8 %)	1 (1. 1 %)	–	–
Chronic kidney disease	1 (0.8 %)	1 (1. 1 %)	–	–
Chronic liver disease	2 (1.7 %)	1 (1. 1 %)	1 (3. 7 %)	0.372
Malignancy	2 (1.7 %)	2 (2. 2 %)	–	–
Others	7 (6.0 %)	3 (3. 4 %)	4 (14. 8 %)	0.030
≥3 comorbidities	4 (3.4 %)	1 (1. 1 %)	3 (11. 1 %)	0.013
Clinical symptoms				
Fever	50 (43.5 %)	37 (42. 0 %)	13 (48. 1 %)	0.576
T>38.0 °C	31 (26.9 %)	23 (26. 1 %)	8 (29. 6 %)	0.720
*T* ≤ 38.0 °C	84 (73.0 %)	65 (73. 8 %)	19 (70. 3 %)	
Cough	44 (38.3 %)	28 (31. 8 %)	16 (59. 2 %)	0.010
Expectoration	30 (26.1 %)	19 (21. 5 %)	11 (40. 7 %)	0.047
Pharyngalgia	34 (29.6 %)	28 (31. 8 %)	6 (22. 2 %)	0.339
Rhinorrhoea	9 (7.8 %)	8 (9. 0 %)	1 (3. 7 %)	0.362
Dyspnea	1 (0.8 %)	–	1 (3.7 %)	–
Fatigue	15 (13.0 %)	10 (11. 3 %)	5 (18. 5 %)	0.334
Myalgia	17 (14.7 %)	14 (15. 9 %)	3 (11. 1 %)	0.539
Nausea	1 (0.8 %)	–	1 (3.7 %)	–
Dysgeusia	1 (0.8 %)	–	1 (3.7 %)	–
Treatment				
Oxygen inhalation	35 (30.4 %)	9 (10. 2 %)	26 (96. 2 %)	<0.001
Chinese traditional medicine	105 (91.3 %)	80 (90. 9 %)	25 (92. 5 %)	0.786
Prone position	28 (24.3 %)	3 (3. 4 %)	25 (92. 5 %)	<0.001
Prophylactic anticoagulation	18 (15.6 %)	1 (1. 1 %)	17 (62. 9 %)	<0.001
Thymosin	19 (16.5 %)	14 (15. 9 %)	5 (18. 5 %)	0.749
Others	21 (18.2 %)	17 (19. 3 %)	4 (14. 8 %)	0.596
Clinical outcomes				
Recovered	115 (100 %)	–	–	–
Hospitalization time (days)	15 (11–19)	15 (13–17)	18 (15–20)	0.017

Abbreviation: BMI= Body Mass Index. Non-Moderate type: including asymptomatic and mild type.

The most common symptoms were fever (50 [43.4 %]), cough (44 [38.2 %]), and pharyngalgia (34 [29.5 %]), followed by expectoration (30 [26.0 %]), myalgia (17 [14.7 %]), fatigue (15 [13.0 %]), rhinorrhoea (9, [7.8 %]), nausea (1 [0.8 %]), dysgeusia (1 [0.8 %]) and dyspnea (1 [0.8 %]). Patients with lower lymphocytes were treated with thymus peptide, and moderate cases received oxygen inhalation, prone position, and prophylactic anticoagulation therapy. All patients recovered with an average hospitalization of 15 days, and the moderate patients were 18 days. However, 6 patients experienced progression during treatment, with new infiltration in the lung, but eventually recovered (Table 1).

Lymphopenia, elevated globulins and elevated blood glucose were the major laboratory abnormalities in these patients. No noteworthy changes were observed in blood flow, liver and renal activity, and cardiac enzymes, probably due to the fact that critically ill patients were not taken into account (Table 2). In moderate patients (*n* = 27), CT demonstrated ground-glass opacities in the peripheral lungs; 63.0 % involved multiple lobes, and the lesions could be unilateral (55.6 %) or bilateral (44.4 %). thickening of the interlobular septa was detected in three cases (Table 3).

**Table 2:** Laboratory test results in patients included in the study.

	All patients (*n* = 115,median)	Non-Moderate type (*n* = 88, median)	Moderate type (*n* = 27, median)	*P*
**Blood routine**				
White blood cell count, × 10^9^/L	6.04 (5.18–7.22)	5.91(5.24–7.03)	6.86 (4.89–7.97)	0.327
Neutrophil count, × 10^9^/L	4.23 (2.94–5.39)	4.17 (2.87–5.35)	4.75 (2.97–5.51)	0.568
Lymphocyte count, × 10^9^/L	1.08 (0.70–1.49)	1.11 (0.75–1.51)	0.94 (0.58–1.38)	0.248
Monocyte count, × 10^9^/L	0.57 (0.42–0.66)	0.55 (0.42–0.64)	0.61 (0.43–0.74)	0.297
Eosinophils count, × 10^9^/L	0.05 (0.02–0.11)	0.05 (0.02–0.09)	0.07 (0.01–0.17)	0.358
Platelet, × 10^9^/L	214 (180–252)	216 (185–256)	180 (147–241)	0.012
**Blood biochemistry**				
Alanine transaminase, U/L	32 (26.0–39.0)	32 (25.0–38.5)	32 (28.0–39.5)	0.636
Aspartate aminotransferase, U/L	25 (21.0–31.0)	24 (20.0–31.0)	27 (23.0–31.5)	0.469
Albumin, g/L	44.4 (41.5–47.3)	44.9 (42.3–47.3)	43 (39.9–47.2)	0.166
Globulin, g/L	33.2 (28.1–38.2)	33.3 (28.1–38.4)	32.8 (28.1–37.3)	0.608
Creatinine, µmol/L	61.6 (48.3–71.4)	61.6 (47.1–69.9)	63.9 (49.3–77.8)	0.394
Lactate dehydrogenase, U/L	219 (179–350)	228 (173–358)	216 (195–311)	0.792
Creatine kinase-isoenzyme, U/L	62 (44.2–93.7)	64 (44.0–94.5)	56 (48.0–85.0)	0.697
Blood glucose, mmol/L	6.2 (5.6–7.0)	6.1 (5.6–7.0)	6.4 (5.9–6.7)	0.507
Serum sodium, mmol/L	138 (135–140)	138 (135–140)	139 (136–141)	0.439
Serum potassium, mmol/L	4.00 (3.84–4.18)	4.05 (3.87–4.18)	3.84 (3.57–4.09)	0.027
Serum calcium, mmol/L	2.29 (2.25–2.36)	2.31 (2.25–2.37)	2.28 (2.23–2.33)	0.207
**Coagulation**				
Prothrombin time, s	11.4 (10.3–12.7)	11.4 (10.3–12.7)	11.3 (10.3–12.4)	0.403
Activated partial thromboplastin time, s	29.6 (26.4–33.9)	29.8 (27.2–33.9)	28.9 (25.2–33)	0.516
D-dimer, µg/L	220 (150–370)	190 (140–350)	320 (180–475)	0.034
Fibrinogen, g/L	2.89 (2.47–3.37)	2.91 (2.47–3.5)	2.85 (2.48–2.99)	0.383
**Infection index**				
C-reactive protein, mg/L	5.5 (2.9–9.1)	5.6 (2.9–9.4)	5.4 (3.3–7.9)	0.946
Interleukin-6, pg/mL	10.0 (9.0–10.6)	9.9 (8.7–10.6)	10.4 (9.3–10.6)	0.068

Note: Non-Moderate type: including asymptomatic and mild type.

**Table 3:** Imaging findings of CT in moderate group (*n* = 27).

	Number ( %)
**Location of the lesion**	
Peripheral	26 (96.3 %)
Central	1 (3.7 %)
Unilateral	15 (55.6 %)
Bilateral	12 (44.4 %)
**Numbers of affected lobes**	
1	10 (37.0 %)
2	9 (33.3 %)
3	6 (22.2 %)
4	1 (3.7 %)
5	1 (3.7 %)
**Imaging features of lesions**	
Ground-glass opacity	8 (29.6 %)
Mottling	19 (70.4 %)
Consolidation	–
Air bronchogram	–
Crazy paving	–
Thickening of interlobular septum	3 (11.1 %)

### 3.2 Ct values and clinical features

The amount of virus in the nasopharyngeal swabs was quantified relative to each other by RT-PCR Ct values.The median Ct value for the ORF 1ab gene was 24.74, according to which we classified the patients into a low Ct value group and a high Ct value group. In the low Ct group, virus shedding took longer, but no statistical difference was observed, probably due to the limited sample size. Patients with low Ct values may have decreased globulins, eosinophils, and elevated leukocytes, monocytes, liver enzymes, creatine kinase isoenzymes, myoglobin, d-dimer, serum sodium, and interleukin-6, which suggests that SARS-CoV-2 Omicron may affect the functioning of the hepatic, immune, cardiac, and coagulation systems (Table 4).

**Table 4:** Clinical and laboratory features in different Ct value group.

	High Ct Value (*n* = 56)	Low Ct Value (*n* = 59)	*P*
Age (years, median)	35 (30–41)	40 (29–64)	<0.001
Gender			
Male	26 (46.4 %)	27 (45. 8 %)	0.943
Female	30 (53.6 %)	32 (54. 2 %)	
Comorbidity	12 (21.4 %)	22 (37. 3 %)	0.062
Vaccination	52 (92.9 %)	42 (71. 2 %)	0.003
Clinical type			
Moderate type	11 (19.6 %)	16 (27. 1 %)	0.344
Non-Moderate type	45 (80.4 %)	43 (72. 9 %)	
Fever	20 (35.7 %)	30 (50. 8 %)	0.102
Cough	18 (32.1 %)	26 (44. 1 %)	0.188
Pharyngalgia	17 (30.4 %)	17 (28. 8 %)	0.856
Negative conversion time (hospitalization days)	14 (12–19)	15 (14–19)	0.087
Blood routine			
White blood cell count, × 10^9^/L	5.82 (5.12–6.81)	6.58 (5.25–7.42)	<0.001
Neutrophil count, × 10^9^/L	4.03 (2.81–5.33)	4.40 (2.97–5.42)	0.033
Lymphocyte count, × 10^9^/L	1.23 (0.73–1.49)	1.03 (0.68–1.49)	0.906
Eosinophils count, × 10^9^/L	0.07 (0.04–0.13)	0.04 (0.01–0.10)	0.036
Monocyte count, × 10^9^/L	0.53 (0.39–0.62)	0.61 (0.47–0.74)	0.062
Blood biochemistry			
Alanine transaminase, U/L	31 (25–38)	33 (28–42)	<0.001
Aspartate aminotransferase, U/L	24 (20–30)	27 (22–32)	<0.001
Globulin, g/L	37.4 (31.9–40.6)	30.5 (26.2–33.5)	<0.001
Lactate dehydrogenase, U/L	346 (207–397)	199 (173–230)	<0.001
Creatine kinase-isoenzyme, U/L	50 (40–71)	72 (54–104)	<0.001
Blood glucose, mmol/L	6.3 (5.8–7)	6.1 (5.5–6.7)	0.390
Serum sodium, mmol/L	135.5 (134.2–138.0)	139.5 (137.2–141.6)	<0.001
Myocardial enzyme			
Myohemoglobin, ng/ml	31.5 (25.1–52.3)	35.7 (27.6–57.0)	<0.001
NT-proBNP, ng/L	29 (15–48)	36 (22–94)	0.021
Coagulation			
D-dimer, µg/L	180 (140–330)	260 (160–430)	<0.001
Fibrinogen, g/L	2.89 (2.46–3.39)	2.85 (2.47–3.36)	0.802
Infection index			
C-reactive protein, mg/L	5.65 (2.42–9.09)	5.40 (3.42–8.87)	0.440
Interleukin-6, pg/ml	9.9 (9.3–10.5)	10.2 (8.9–10.7)	<0.001
			

Abbreviation: NTproBNP = *N*-terminal pro-B-type natriuretic peptide.

### 3.3 Predictors of low Ct value

We further assessed the effect of various factors on low Ct values in these patients. By univariate analysis, age, comorbidities, vaccination, globulin, serum sodium and monocyte count were significantly associated with low Ct values. The results showed that elevated monocyte count (OR: 3.556; 95 % CI: 1.330–9.503) was associated with low Ct values. Vaccination within one year (OR: 0.209; 95 % CI: 0.051–0.854) and lower serum sodium (OR: 0.137; 95 % CI: 0.051–0.367) were negatively associated with low Ct values (Table 5).

**Table 5:** Univariate and multivariate analysis of risk factors for lower Ct value (high viral load) group.

Risk Factor	Univariate analysis			Multivariate analysis		
	OR	95 % CI	*P*	OR	95 % CI	*P*
Patient age, y (≥60 vs <60)	5.231	1.8031–5.173	0.002	2.919	0.719–11.843	0.134
Comorbidity (Yes vs No)	2.180	0.952–4.991	0.065	1.069	0.297–3.848	0.919
Vaccination (Yes vs No)	0.190	0.059–0.608	0.005	0.209	0.051–0.854	0.029
Monocyte count (Elevated vs Normal)	2.540	1.182–5.460	0.017	3.556	1.330–9.503	0.011
Globulin (Elevated vs Normal)	0.212	0.088–0.212	0.001	0.383	0.138–1.059	0.064
Serum sodium (Decreased vs Normal)	0.111	0.047–0.261	<0.001	0.137	0.051–0.367	˂0.001

## 4 Discussion

In our study, Ct value presented viral load which was not only a diagnostic tool, but may also offer benefit to clinicians in making clinical decisions. The Ct value, a key indicator in reverse transcription polymerase chain reaction (RT-PCR) assays, reflects the abundance of viral RNA in a sample and thus indirectly measures viral load (Paltiel et al., 2020). Ct value were associated with monocyte count, vaccination state and serum sodium. Lower Ct values usually imply higher viral loads, and this information has multiple implications for clinical treatment and management. First, a low Ct value can be a marker of early infection or high infectiousness, prompting clinicians to monitor and manage patients more closely, for example, with earlier use of antiviral medications, increased isolation, and close observation of the risk of possible clinical deterioration. Secondly, regular monitoring of Ct values can help to assess treatment efficacy and disease progression, and provide a basis for timely adjustment of treatment regimens. Finally, combining Ct with other clinical parameters (e.g., symptom severity, inflammation indicators, etc.) is expected to lead to the construction of a comprehensive risk assessment model for individualised treatment and precise management. Future studies need to further define the optimal clinical threshold of Ct and its association with patient prognosis, in order to provide a more accurate and scientific basis for clinical decision-making.Low Ct values (high viral load) presented worse laboratory results of blood routine, liver and coagulation system.Some studies on viral load have confirmed that severe patients of COVID-19 tend to have a high viral load and a long virus-shedding aperiod (Liu et al., 2020; Huang et al., 2020). Low Ct values correlated with increased probability of progression to severe disease, increased disease severity and increased mortality (Rao et al., 2020). If the Ct value was lower, the lymphocyte count was lower, organ damage was greater, and the time it took to turn negative was longer (Liu et al., 2020; Yin et al., 2021; Chen et al., 2021).

Elevated globulin facilitates recovery from infection with the SARS-CoV-2 omicron variant, which activates humoral immunity and specific immunoglobulins (e.g., IgG and IgM) to clear the virus. Patients with low globulins tend to have high viral loads, suggesting that globulins may be a protective factor against viral load. Monocytes in COVID-19 were the first element involved in innate immunity (Merad and Martin, 2020). As reported, monocyte distribution width was significantly higher in patients with COVID-19, independently distinguishing COVID-19 from influenza (Lin et al., 2020). A strong innate response, characterized by mobilization of activated CD14^+^ CD16^+^ monocytes during the first days of infection, was detectable even in patients with mild disease (Vetter et al., 2020). This is consistent with our result that monocyte count elevated in high viral load of SARS-CoV-2 cases. Monocyte biology appears considerably activated in patients with SARS‐CoV‐2 infection (Frater et al., 2020). This may be due to the direct cytopathic effect of SARS‐CoV‐2 on this cell lineage, as well as to direct or indirect cell activation by circulating cytokines or immunocomplexes (Lippi et al., 2021).

The count of lymphocytes and eosinophils was reduced in COVID-19 patients. Lymphopenia is more accentuated in symptomatic patients with pneumonia compared with those without pneumonia (de Candia et al., 2021; Pan et al., 2022). A substantial decrease in the number of lymphocytes indicates that the coronavirus consumes immune cells and inhibits the body's cellular immune function (Gu et al., 2022). Damage to T lymphocytes might be an important factor inducing exacerbations of patients (Liu et al., 2017). Eosinophils develop from pluripotent hematopoietic stem cells and were not reported infiltrating the target organ on autopsy. However, previous studies have suggested that viral infection can directly inhibit bone marrow function and reduce eosinophil production (Perng, 2012), and we speculates that the omicron may also have direct inhibition of bone marrow function resulting in decreased eosinophil.

Studies have reported electrolyte disorders such as hypokalemia and hyponatremia (Lippi et al., 2020). We observed that a potential association between serum sodium levels and Ct values, and although this phenomenon has been partially discussed in previous studies, the exact mechanism remains unclear. Serum sodium, an important physiological indicator, is known to play a key role in the maintenance of cellular electrolyte balance, osmotic pressure, and neuromuscular function (Chen and Xu, 2019). Low serum sodium levels may affect the functioning of the immune system, which in turn alters the body's response to viral infection, thereby affecting the results of viral load assays (Peng et al., 2023; Rabaan et al., 2021). However, the current study did not delve into the biological mechanisms underlying the association between low serum sodium and lower Ct values.To further understand this observation, future studies need to delve into how lower serum sodium may lead to lower Ct values by modulating pathways such as the immune system, cellular metabolism, or viral replication. This process may involve the role of sodium ions in immune cells, dysfunction of the sodium-potassium pump or correlation with the inflammatory response. We suggest exploring the biological mechanisms behind this phenomenon through more clinical and laboratory studies that incorporate trends in serum sodium levels and Ct values to provide new perspectives for future predictive models and therapeutic strategies.

SARS-CoV-2 infection leads to pathological changes in systemic metabolism. We have found the phenomenon of hyperglycemia regardless of whether the patient has diabetes or not. Studies have found COVID-19 might predispose infected individuals to hyperglycaemia and increase the severity of COVID-19 in patients with diabetes mellitus. Hyperglycemia interacts with other risk factors and may modulate immune and inflammatory responses, making patients vulnerable to severe COVID-19 and possible fatal outcome (Lim et al., 2021). Scientists have detected altered glycometabolic control, insulin resistance and an abnormal cytokine profile in COVID-19 patients and glycaemic abnormalities can be detected for at least 2 months in patients who recovered from COVID-19 (Montefusco et al., 2021).Recent studies have shown that GP73 is a gluconeogenic hormone that contributes to SARS-CoV-2-induced hyperglycaemia by promoting hepatic gluconeogenesis. (Wan et al., 2022).

The cases in the present study were probably due to early detection and treatment, and there were no critical cases among them, unlike the ancestral strain in Wuhan. Patients had mild upper respiratory symptoms and some had pulmonary infiltrates. Once infected with the Omicron variant, 48.5 percent of patients had fever and 38.3 percent had cough, which was much less frequent than the Delta variant. This is consistent with the results of other studies that have shown that the Omicron variant has a lower risk of developing serious clinical outcomes, including hospital admission, admission to the intensive care unit, mechanical ventilation, and death, compared with the Delta variant (Wang et al., 2021).

The study allows us to draw some lessons that are useful for clinical selection. For older, unvaccinated and sicker patients, strict care should be given, such as adopting the prone position as early as possible (Xu et al., 2022). For confirmed patients of omicron, we should pay more attention to the patient's blood glucose and electrolytes, and correct abnormalities in time. Abnormally elevated monocytes during treatment may indicate the patient is still under high viral load. The value of Ct values lies in the information we can obtain from each patient, which is significant not only in terms of viral load and diagnostic criteria, but also in the fact that we can obtain additional clinical information that can help us to understand the evolution of the disease and to manage patients with COVID-19 patients in a patient management way, as well as to guide infection control, public health and occupational health decisions.

## 5 Limitations

Our study, however, has certain restrictions. we used a single-centre design and a small cohort, limiting the applicability of the results to a wider population. Due to regional differences in sample sources, there may be some regional and demographic differences, particularly in the impact of different SARS-CoV-2 variants in the context of their prevalence. Therefore, future studies should consider multicentre, large-scale cohort studies to better assess the prevalence and applicability in different regions and populations. In addition, the cross-sectional study design of this paper also failed to delve into long-term follow-up and potential causality, and subsequent longitudinal studies will help to further validate these results. Most patients were unresponsive, mild and moderate, and there was scant data on severe and critical sickness. Although we have strict quality control standards, there is inconsistency of the Ct values which may vary across different assays and instruments.

## 6 Conclusions

The omicron variant mainly impacts on lymphocyte, globulin, and blood glucose. Patients with low Ct values are mostly elderly and unvaccinated. We identified several clinical predictors associated with Ct values, such as monocytes, vaccination, and serum sodium, which may suggest the potential of Ct values as a clinical predictor, and may also provide some assistance to clinicians and health policy makers in the fight against epidemics.

## Ethics approval and consent to participate

This study was approved by the Ethics Committee of the First Affiliated Hospital of Soochow University. Informed consent was obtained from all the participants. All methods were carried out in accordance with Declaration of Helsinki.

## Clinical trial number

Not applicable.

## Consent for publication

Not Applicable.

## Availability of data and material

The datasets used and analysed during the current study available from the corresponding author on reasonable request.

## Funding

This work was supported by Jiangsu Provincial Medical Key Discipline (ZDXK202201), Gusu Key Medical Talent Program (grant No. GSWS2020017, GSWS2020093) and Youth Scientific Research Project of Suzhou Health Commission, China (KJXW2019045).

## CRediT authorship contribution statement

**Wen Yang:** Writing – original draft, Resources, Methodology, Formal analysis, Conceptualization. **Tao Tao:** Resources, Data curation. **Jianping Zhang:** Software, Formal analysis. **Yuting Yao:** Supervision. **Min Chen:** Visualization. **Mingming Liu:** Supervision. **Meiying Wu:** Writing – review & editing, Validation, Investigation. **Wei Lei:** Writing – review & editing, Software, Project administration, Funding acquisition.

## Declaration of competing interest

The authors declare that they have no known competing financial interests or personal relationships that could have appeared to influence the work reported in this paper.

## Data Availability

Data will be made available on request.

## References

[bib0001] Cascella M., Rajnik M., Aleem A., Dulebohn S.C., Di Napoli R. (2022). StatPearls.

[bib0005] Chen Yulin, Xu Wei (2019). Hyponatremia and hyponatremic encephalopathy in children. Chin. Pediatr. Emerg. Med..

[bib0004] Chen W., Xiao Q., Fang Z., Lv X., Yao M., Deng M. (2021). Correlation analysis between the viral load and the progression of COVID-19. Comput. Math. Methods Med..

[bib0003] Chen D.-Y., Chin C.V., Kenney D. (2023). Spike and nsp6 are key determinants of SARS-CoV-2 Omicron BA.1 attenuation. Nature.

[bib0006] Chu C.M., Poon L.L.M., Cheng V.C.C. (2004). Initial viral load and the outcomes of SARS. CMAJ.

[bib0007] de Candia P., Prattichizzo F., Garavelli S. (2021). T cells: warriors of SARS-CoV-2 infection. Trends. Immunol..

[bib0008] Diagnosis and Treatment Protocol for Novel Coronavirus Pneumonia (Trial Version 8). 2022. Available at: http://www.nhc.gov.cn/yzygj/s7653p/202104/7de0b3837c8b4606a0594aeb0105232b.shtml. 10.1097/CM9.0000000000000819 PMC7213636 32358325

[bib0009] Frater J.L., Zini G., d'Onofrio G., Rogers H.J. (2020). COVID-19 and the clinical hematology laboratory. Int. J. Lab. Hematol..

[bib0010] Gu W., Gan H., Ma Y. (2022). The molecular mechanism of SARS-CoV-2 evading host antiviral innate immunity. Virol. J..

[bib0012] Huang J.T., Ran R.X., Lv Z.H. (2020). Chronological changes of viral shedding in adult inpatients with COVID-19 in Wuhan, China. Clinic. Infect. Dis..

[bib0013] Lim S., Bae J.H., Kwon H.-S. (2021). COVID-19 and diabetes mellitus: from pathophysiology to clinical management. Nat. Rev. Endocrinol..

[bib0014] Lin H.A., Lin S.F., Chang H.W. (2020). Clinical impact of monocyte distribution width and neutrophil-to-lymphocyte ratio for distinguishing COVID-19 and influenza from other upper respiratory tract infections: a pilot study. PLoS ONE.

[bib0016] Lippi G., South A.M., Henry B.M. (2020). Electrolyte imbalances in patients with severe coronavirus disease 2019 (COVID-19). Ann. Clin. Biochem..

[bib0015] Lippi G., Sanchis-Gomar F., Henry B.M. (2021). Pooled analysis of monocyte distribution width in subjects with SARS-CoV-2 infection. Int. J. Lab. Hematol..

[bib0017] Liu W.J., Zhao M., Liu K. (2017). T-cell immunity of SARS-CoV: implications for vaccine development against MERS-CoV. Antiviral Res..

[bib0018] Liu Y., Liao W., Wan L., Xiang T., Zhang W. (2020). correlation between relative nasopharyngeal virus RNA load and lymphocyte count disease severity in patients with COVID-19. Viral. Immunol..

[bib0019] Liu Y., Yan L., Wan L. (2020). Viral dynamics in mild and severe cases of COVID-19. Lancet Infect. Dis..

[bib0020] Merad M., Martin J.C. (2020). Pathological inflammation in patients with COVID-19: a key role for monocytes and macrophages. Nat. Rev. Immunol..

[bib0021] Montefusco L., Ben Nasr M., D'Addio F. (2021). Acute and long-term disruption of glycometabolic control after SARS-CoV-2 infection. Nat. Metab..

[bib0022] Paltiel A.D., Zheng A., Walensky R.P. (2020). Assessment of SARS-CoV-2 screening strategies to permit the safe reopening of college campuses in the United States. JAMA Netw. Open..

[bib0023] Pan P., Du X., Zhou Q. (2022). Characteristics of lymphocyte subsets and cytokine profiles of patients with COVID-19. Virol. J..

[bib0024] Peng S., Peng J., Yang L., Ke W. (2023). Relationship between serum sodium levels and all-cause mortality in congestive heart failure patients: a retrospective cohort study based on the Mimic-III database. Front. Cardiovasc. Med..

[bib0025] Perng G.C. (2012). Role of bone marrow in pathogenesis of viral infections. J. Bone Marrow Res..

[bib0026] Rabaan A.A., Tirupathi R., Sule A.A. (2021). Viral dynamics and real-time RT-PCR ct values correlation with disease severity in COVID-19. Diagnostics.

[bib0027] Rao S.N., Manissero D., Steele V.R. (2020). A systematic review of the clinical utility of cycle threshold values in the context of COVID-19. Infect. Dis. Ther..

[bib0028] Vetter P., Eberhardt C.S., Meyer B. (2020). Daily viral kinetics and innate and adaptive immune response assessment in COVID-19: a case series. mSphere.

[bib0029] Wan L., Gao Q., Deng Y. (2022). GP73 is a glucogenic hormone contributing to SARS-CoV-2-induced hyperglycemia. Nat. Metab..

[bib0030] Wang Y., Chen R., Hu F. (2021). Transmission, viral kinetics and clinical characteristics of the emergent SARS-CoV-2 Delta VOC in Guangzhou, China. EClinicalMedicine.

[bib0002] Xu C.-C., Xu J.-l., Wang X.-f. (2022). Prone position reduces the risk of patients with mild or moderate COVID-19 progressing to severe or even critical cases: a retrospective study. Eur. J. Med. Res..

[bib0031] Yin S.-W., Zhou Z., Wang J.-L., Deng Y.-F., Jing H., Qiu Y. (2021). Viral loads, lymphocyte subsets and cytokines in asymptomatic, mildly and critical symptomatic patients with SARS-CoV-2 infection: a retrospective study. Virol. J..

